# Inhibition of calcium-independent phospholipase A impairs agonist-induced calcium entry in keratinocytes

**DOI:** 10.1111/j.1365-2133.2007.08298.x

**Published:** 2008-01

**Authors:** K Ross, G Parker, M Whitaker, NJ Reynolds

**Affiliations:** Dermatological Sciences, Institute of Cellular Medicine, Medical School, Newcastle UniversityNewcastle upon Tyne NE2 4HH, U.K.; * Institute of Cell and Molecular Biosciences, Medical School, Newcastle UniversityNewcastle upon Tyne NE2 4HH, U.K.

**Keywords:** calcium entry, calcium-independent phospholipase A, keratinocytes

## Abstract

**Background:**

In many cells, depletion of intracellular calcium (Ca^2+^) reservoirs triggers Ca^2+^ entry through store-operated Ca^2+^ channels in the plasma membrane. However, the mechanisms of agonist-induced calcium entry (ACE) in keratinocytes are not fully understood.

**Objectives:**

This study was designed to determine if pharmacological inhibition of calcium-independent phospholipase A (iPLA_2_) impairs ACE in normal human epidermal keratinocytes.

**Methods:**

Confocal laser scanning microscopy was used to monitor the dynamics of Ca^2+^ signalling in keratinocytes loaded with the calcium-sensitive dye Fluo-4. Cells were stimulated with extracellular nucleotides [adenosine triphosphate (ATP) or uridine triphosphate (UTP)] or with lysophosphatidic acid (LPA), a bioactive lipid that regulates keratinocyte proliferation and differentiation.

**Results:**

Both ATP and UTP induced Ca^2+^ release in primary human keratinocytes. This was not followed by robust Ca^2+^ influx when the experiments were performed in low Ca^2+^ (70 μmol L^−1^) medium. Upon elevation of extracellular Ca^2+^ to 1·2 mmol L^−1^, however, a biphasic response consisting of an initial Ca^2+^ peak followed by an elevated plateau was observed. The plateau phase was inhibited when cells were treated with bromoenol lactone, a specific pharmacological inhibitor of iPLA_2_. These findings indicate that iPLA_2_ activity is required for ACE in keratinocytes. LPA also evoked Ca^2+^ release in keratinocytes but failed to induce sustained Ca^2+^ entry even when extracellular Ca^2+^ was elevated to 1·2 mmol L^−1^.

**Conclusion:**

Our results demonstrate for the first time an important role for iPLA_2_ in regulating ACE in primary human keratinocytes.

Calcium is a ubiquitous second messenger that regulates numerous cellular processes such as gene transcription, cell proliferation, exocytosis and contraction.^1^ Free cytosolic calcium ([Ca^2+^]_i_) levels are tightly controlled by a complex network of receptors, channels and pumps located in the plasma membrane (PM) and on intracellular organelles such as the endoplasmic reticulum (ER), mitochondria and the Golgi apparatus. Stimulation of G protein-coupled receptors (GPCRs), tyrosine kinase receptors and nonreceptor tyrosine kinases activate phospholipase C (PLC) which in turn hydrolyses phosphatidylinositol 4,5-bisphosphate to diacylglycerol and inositol 1,4,5-trisphosphate (IP_3_).^2^ Binding of IP_3_ to its receptors (IP_3_R) on the ER triggers the release of Ca^2+^ from the ER lumen leading to store depletion.^1^ In many cells, this initial release is followed by a sustained influx of Ca^2+^ across the PM, a phenomenon known as store-operated calcium entry (SOCE), which is the dominant form of Ca^2+^ entry in nonexcitable cells.^3^

One model of SOCE involves a diffusible messenger or ‘calcium influx factor’ (CIF) that is released from the ER upon store depletion.^4^ Although the identity of CIF is unknown, it appears to be a soluble factor of 600 Da that activates calcium-independent phospholipase A (iPLA_2_) by displacement of inhibitory calmodulin (CaM) from iPLA_2_.^5^ This leads to the production of lysophospholipids (lysoPLs) and free fatty acid. The lysoPLs, such as lysophosphatidylcholine, then activate SOCE at the PM by an uncharacterized process. Thus iPLA_2_ activity appears to be required for SOCE.

A second model for SOCE has emerged recently, involving STIM1 and Orai1. STIM1, a Ca^2+^-sensing protein localized predominantly to the ER contains a low-affinity Ca^2+^-binding EF hand which resides in the ER lumen when the stores are full. Depletion of the stores by IP_3_-mediated Ca^2+^ release, or by inhibition of the sarco-endoplasmic reticulum Ca^2+^-ATPase (SERCA) pump with thapsigargin (TG) causes Ca^2+^ to dissociate from STIM1 inducing the re-organization of STIM1 into discrete puncta.^6^,^7^ These complexes appear to associate with, and activate, the transmembrane protein Orai1, which appears to be the pore through which SOCE occurs.^8^–^12^ Interestingly, STIM1 has also been reported to activate TRPC1,^13^,^14^ a member of the transient receptor potential (TRP) family of proteins which have been implicated in cation entry.^15^

Little is known about the mechanisms of Ca^2+^ entry in keratinocytes. A requirement for PLCγ for SOCE has been demonstrated,^16^ along with the formation of a ternary complex composed of PLCγ, TRPC1 and IP_3_R. However, the putative role of iPLA_2_ in Ca^2+^ entry in keratinocytes has not been examined. In the present study therefore, we have investigated the role of iPLA_2_ in agonist-induced Ca^2+^ entry (ACE) in normal human epidermal keratinocytes (NHEKs). We have performed our studies using physiological agonists such as adenosine triphosphate (ATP) and uridine triphosphate (UTP), which have been reported to promote NHEK proliferation *in vitro*^17^,^18^ and lysophosphatidic acid (LPA, 1-acyl-*sn*-glycerol-3-phosphate), which can promote proliferation or differentiation depending on cell density.^19^ These agonists are released by platelets recruited to the epidermis following injury,^17^,^20^ indicating a role in epidermal homeostasis. We found that the extracellular nucleotides but not LPA evoked sustained ACE in NHEK and that this was mediated at least in part by iPLA_2_.

## Materials and methods

### Reagents

Fluo-4-AM was purchased from Molecular Probes (Eugene, OR, U.S.A.), bromoenol lactone (BEL) from Sigma (Poole, Dorset, U.K.) and the iPLA_2_ antibody from Santa Cruz (Santa Cruz, CA, U.S.A.). The iPLA_2_ antibody recognizes iPLA2β but not iPLA_2_γ according to the manufacturer. All other reagents were obtained from Sigma unless stated otherwise.

### Cell culture

NHEK were prepared from redundant foreskin with the approval of the Newcastle and North Tyneside local ethical committee. The cells were cultured in supplemented MCDB 153 culture medium as previously described.^21^ For imaging, cells were seeded at passage 1 or 2 in 20 μL suspensions containing 10 000–20 000 cells, with the medium increased to 1 mL about 45 min to 1 h after seeding.

### Calcium imaging

Subconfluent monolayers of cells, seeded in Willco glass-bottomed microwell dishes (Intracel, Royston, U.K.) 1 day before experiments, were loaded with 3 μmol L^−1^ of Fluo-4 acetoxymethyl (AM) ester (Molecular Probes) for 45 min at 37 °C. Dye loading and all subsequent steps were performed with MCDB153 medium (Sigma) containing 70 μmol L^−1^ Ca^2+^ unless indicated otherwise. To minimize uptake of the dye into organelles, 200 μmol L^−1^ of the anion transport inhibitor sulphinpyrazone was dissolved in dimethyl sulphoxide (DMSO) and included in the medium during loading and de-esterification. After loading, the cells were washed in Ca^2+^ and Mg^2+^ free phosphate-buffered saline (PBS) and incubated in MCDB 153 medium with 70 μmol L^−1^ Ca^2+^ for 1 h at 37 °C to allow de-esterification of the intracellular dye. Where indicated, Ca^2+^ in the medium was raised to 1·2 mmol L^−1^ at the start of the de-esterification phase. The iPLA_2_ inhibitor BEL (10 or 20 μmol L^−1^) or vehicle (0·1% DMSO) was added for the last 30 min of de-esterification.

The cells were maintained at 37 °C during image acquisition with a heated stage. Changes in [Ca^2+^]_i_ were detected with a Leica TCS SP2 confocal laser scanning microscope equipped with an argon laser (Leica, Milton Keynes, U.K.). Fluorescence excitation was performed with the 488 nm line of the laser. Fluorescence emission was collected through a 500–550 nm window of the detector. Images were captured with a 63X Plan Apo objective (NA1.32) at 4-s intervals as 12-bit frames of 512 × 512 pixels. The perimeter of each cell was outlined to define the region of interest whose mean fluorescence intensity in each frame was determined by Leica confocal software. The changes in [Ca^2+^]_i_ were expressed as the ratio of the temporal fluorescence to the initial fluorescence (Ft/F0).

### Western blotting

Primary keratinocyte lysates were separated on a 10% Bis-Tris gel (Invitrogen, Paisley, U.K.), transferred to a nitrocellulose membrane and incubated overnight with 10 μg mL^−1^ of iPLA_2_ antibody (goat polyclonal, Santa Cruz). After extensive washing the membrane was probed with a biotinylated secondary antibody for 2–3 h, processed for chemiluminescence using ABC reagents (Vector Laboratories, Burlingame, CA, U.S.A.) and ECL Advance™ (GE Healthcare, Little Chalfont, U.K.), then visualized on a phosphoimager.

### Statistical analysis

Results of the Ca^2+^ imaging experiments are presented as means (± SEM) which were determined in GraphPad Prism (GraphPad Software, San Diego, CA, U.S.A.) or Microsoft Excel. Statistical significance was calculated using the unpaired two-tailed Student’s *t*-test. A *P*-value < 0·05 was considered significant.

## Results

### Agonist-induced Ca^2+^ entry in primary keratinocytes

**Fig 1:**
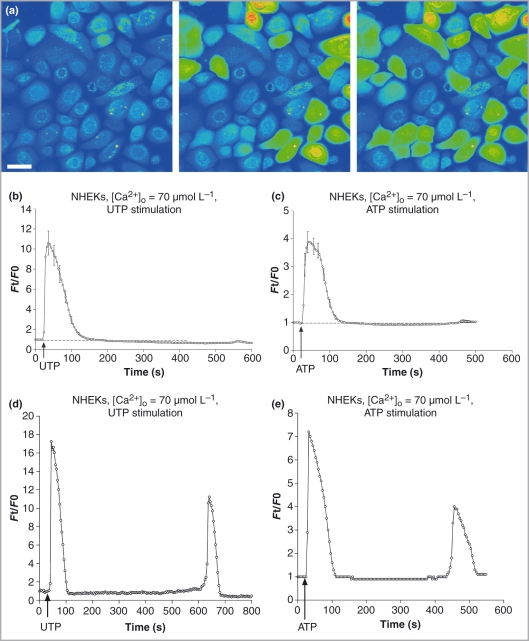
Ca^2+^ mobilization in normal human epidermal keratinocytes (NHEKs) following uridine triphosphate (UTP) or adenosine triphosphate (ATP) stimulation. (a) Pseudocolour confocal micrographs of NHEKs loaded with Fluo-4 and stimulated with 10 μmol L^−1^ UTP. The images shown are for t = 20 s (that is, just prior to addition of the agonist), t = 28 s and t = 40 s. Scale bar, 47 μm. (b,c) Averaged changes in Fluo-4 intensity following stimulation of NHEKs in medium containing 70 μmol L^−1^ calcium with 10 μmol L^−1^ UTP or ATP. The agonists were added 20 s after the start of recording, as indicated by the arrow. The data shown for each agonist were pooled from three independent experiments (cell preparations from three independent donors), with *n*=59 cells and *n*=32 cells for (b) and (c), respectively. For clarity, only selected error bars are shown. Error bars = SEM. (d,e) Representative traces from cells in which a second [Ca^2+^]_i_ peak was observed after the initial peak evoked by UTP (d) or ATP (e) stimulation.

Several studies have shown that ATP and UTP evoke [Ca^2+^]_i_ elevation in primary keratinocytes,^17^,^22^ but the extent to which this promotes a sustained increase in [Ca^2+^]_i_ (indicative of Ca^2+^entry) was not clear. When preconfluent monolayers of NHEKs were stimulated with UTP, a single [Ca^2+^]_i_ transient was observed in the majority of responsive cells (Fig. 1a,b). The rise in [Ca^2+^]_i_ began immediately following the addition of the agonist, and reached a peak about 16 s thereafter. The entire [Ca^2+^]_i_ signal lasted just over 2 min before returning to baseline levels. In 6% (6 of 98 responsive cells from four experiments), a second transient of smaller amplitude was observed at later time points (Fig. 1d). Stimulation of the cells with ATP also induced a single 2 min [Ca^2+^]_i_ transient in most of the responsive cells (Fig. 1c), with a second [Ca^2+^]_i_ peak in 23% of the cell population (16 of 70 responsive cells pooled from five experiments; Fig. 1e).

**Fig 2:**
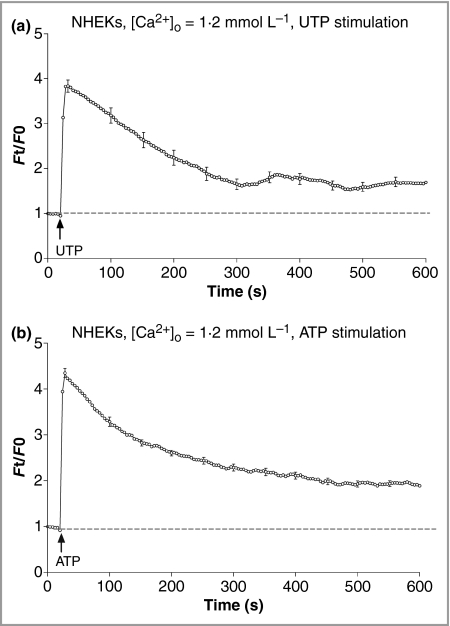
Agonist-induced calcium entry (ACE) in normal human epidermal keratinocytes (NHEKs) occurs upon elevation of extracellular Ca^2+^. NHEKs were switched to MCDB 153 medium containing 1·2 mmol L^−1^ Ca^2+^ for at least 1 h and stimulated with 10 μmol L^−1^ of each agonist as indicated. Averaged time courses of the [Ca^2+^]_i_ signal for (a) UTP (*n*=23 cells) and (b) ATP (*n*=24 cells). Data shown are representative of at least three independent experiments (cell preparations from three independent donors), that gave similar results. For clarity, only selected error bars are shown. Error bars = SEM.

In these experiments, which were performed in medium containing 70 μmol L^−1^ Ca^2+^ we consistently failed to observe sustained [Ca^2+^]_i_ elevation. We therefore repeated the assays after switching to medium containing 1·2 mmol L^−1^ [Ca^2+^]_o_ for ∼1 h. Over several hours 1 mmol L^−1^ extracellular calcium itself stimulates a significant rise in [Ca^2+^]_i_ and promotes differentiation.^23^ Both UTP and ATP induced a biphasic response under these conditions, with a defined initial peak followed by an elevated plateau indicative of Ca^2+^entry (Fig. 2a,b). Taken together, these results indicate that the extracellular calcium ([Ca^2+^]_o_) level needs to be in the millimolar range before the electrochemical gradient is sufficiently high to promote robust ACE in keratinocytes following stimulation with exogenous nucleotides.

### Inhibition of iPLA_2_ impairs agonist-induced calcium entry in primary keratinocytes

**Fig 3:**
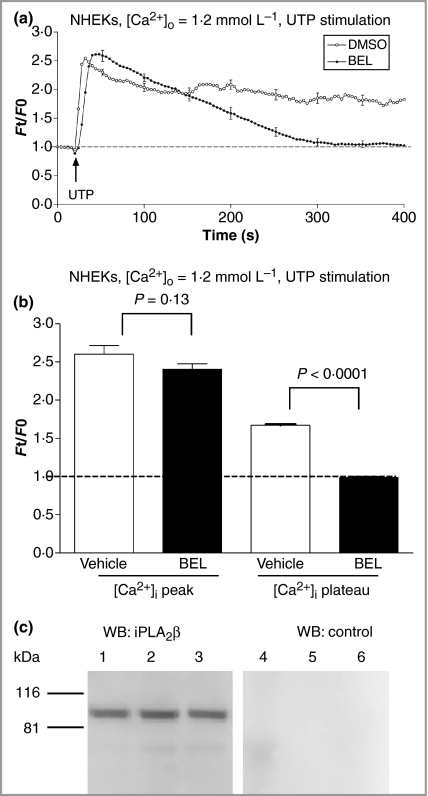
Inhibition of iPLA_2_ impairs agonist-induced calcium entry (ACE) in normal human epidermal keratinocytes (NHEKs). (a) Extracellular calcium was raised to 1·2 mmol L^−1^ for at least 1 h before image acquisition. Changes in the Ft/F0 ratio averaged from paired assays on NHEKs treated with the iPLA_2_ inhibitor bromoenol lactone (BEL) for 30 min (filled circles, *n*=8), or with vehicle (DMSO, open circles, *n*=18) prior to stimulation with 10 μmol L^−1^ uridine triphosphate (UTP). Representative data are from three independent experiments (cell preparations from three independent donors). (b) Summary data for abrogation of UTP-induced Ca^2+^ entry following inhibition of iPLA_2_. Data were pooled from three independent experiments that gave similar results. Plateau phases were averaged over 100 s in each case. DMSO, *n*=54 cells; BEL, *n*=63 cells. Error bars = SEM. (c) Western blotting confirmed the presence of iPLA_2_ in NHEK. An 85-kDa band corresponding to iPLA_2_ was detected in lysates from untreated (lane 1), lithium-treated (10 mmol L^−1^, 2 days; lane 2) and calcium-treated (1·5 mmol L^−1^, 2 days; lane 3) NHEKs probed with an antibody against iPLA_2_ (left panel). No band was seen if the iPLA_2_ antibody was excluded from the assay (right panel).

A recent study by Bolotina and co-workers showed that pharmacological inhibition or RNAi knockdown of iPLA_2_ impaired TG-induced entry in mouse smooth muscle cells.^4^ Although the TG-induced SOCE has historically been considered as a mechanistic parallel of ACE, differences are beginning to emerge.^24^,^25^ Thus, we asked if iPLA_2_ activity was required for ACE in NHEKs. For these studies, we used the suicide substrate, BEL, to inhibit iPLA_2_ activity. BEL is a specific inhibitor of iPLA_2_, with a 1000-fold selectivity for iPLA_2_ over cytosolic PLA_2_.^26^ Paired assays were performed on keratinocytes from the same donors. As shown in Figure 3, stimulation with UTP in the presence of 1·2 mmol L^−1^ [Ca^2+^]_o_ led to a sustained [Ca^2+^]_i_ plateau in control cells exposed to vehicle (DMSO), consistent with the results shown in Figure 2a. In contrast, the elevated [Ca^2+^]_i_ phase was impaired in cells treated with BEL (Fig. 3a), returning to baseline by 300 s in contrast to control cells. It is important to note that the control cells in Figure 3a were treated with dimethylsulphoxide which might explain why their responses differed somewhat to those in Figure 2a. Nevertheless, the data suggest that BEL treatment impairs Ca^2+^entry in NHEK. Furthermore, similar results were obtained on the HaCaT keratinocyte cell line.^39^ The expression of iPLA_2_ in NHEK was assessed by Western blotting (Fig. 3c). An 85-kDa band corresponding to the expected size of iPLA_2_ was specifically detected in NHEK lysates probed with an antibody against iPLA_2_ confirming that iPLA_2_ is expressed in these cells.

### LPA-induced [Ca^2+^]_i_ signalling in keratinocytes

**Fig 4:**
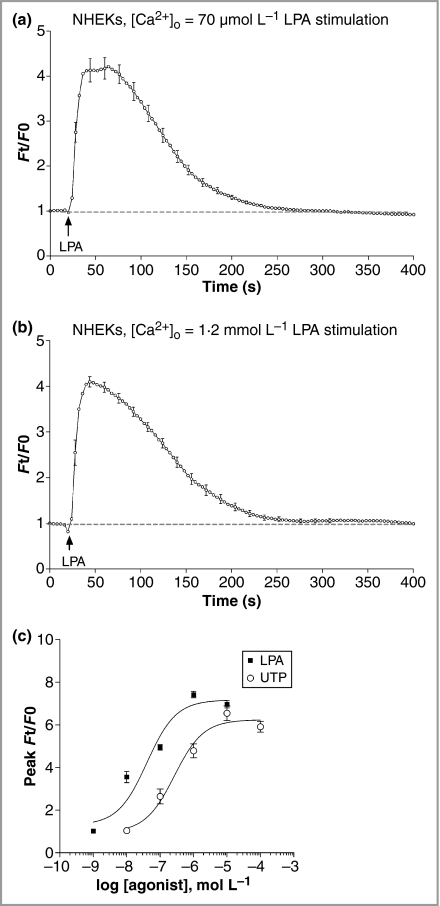
Lysophosphatidic acid (LPA) induces [Ca^2+^]_i_ release but not sustained [Ca^2+^]_i_ elevation. Normal human epidermal keratinocytes (NHEKs) were loaded with Fluo-4 and stimulated with 10 μmol L^−1^ LPA 20 s after the start of recording, as indicated by the arrow. (a) Averaged changes in Fluo-4 intensity following LPA stimulation (*n*=94 cells). Data were pooled from three independent experiments (cell preparations from three independent donors). (b) Extracellular calcium was raised to 1·2 mmol L^−1^ for 1 h before stimulation with 10 μmol L^−1^ LPA (*n*=34 cells). Three independent experiments gave similar results. (c) Dose–response curves for LPA and uridine triphosphate (UTP)-induced [Ca^2+^]_i_ elevation in NHEKs at 37 °C. The peak of the Ft/F0 ratio attained in each cell was determined for each concentration of agonist. Results are the means ± SEM for 19–52 cells.

Intracellular Ca^2+^ release by UTP and ATP is mediated by the P2Y family of GPCRs.^18^ To determine if stimulation of a different class of PLCβ-activating GPCRs also evoked ACE in keratinocytes, we examined the [Ca^2+^]_i_ dynamics induced by LPA. This bioactive phospholipid has been shown to modulate keratinocyte growth and differentiation^19^ and signals through members of the LPA_1–3_ family of GPCRs. In NHEK maintained in medium containing 70 μmol L^−1^ [Ca^2+^]_o_, LPA (10 μmol L^−1^) evoked a single [Ca^2+^]_i_ transient that reached a peak about 20 s thereafter (Fig. 4a). The peak [Ca^2+^]_i_ signal persisted for about 30 s before starting to decline to resting levels. Overall the transient lasted for about 4 min, significantly longer than that observed with extracellular nucleotides. We then asked if elevation of [Ca^2+^]_o_ to 1·2 mmol L^−1^ would facilitate the induction of an elevated [Ca^2+^]_i_ plateau following LPA stimulation. In contrast to ATP and UTP, the LPA-induced [Ca^2+^]_i_ transient produced under these conditions was not generally followed by a [Ca^2+^]_i_ plateau (Fig. 4b). This result also controls against the possibility that raised (1·2 mmol L^−1^) [Ca^2+^]_o_ was exerting a nonspecific effect enhancing effect on ACE in keratinocytes. In addition, the duration of the transient was not significantly altered by the inclusion of 10 mmol L^−1^ EGTA in the medium, indicating that sustained Ca^2+^ entry did not occur (data not shown). Thus LPA does not appear to induce significant Ca^2+^ influx in keratinocytes. This observation is consistent with the findings of others on T cells and fibroblasts.^27^,^28^ The inability of LPA to evoke ACE in our experiments was not due to submaximal stimulation because dose–response curves revealed that LPA-mediated Ca^2+^ release peaked at about 1 μmol L^−1^ (Fig. 4c). Furthermore, LPA was a more potent inducer of Ca^2+^ release than UTP, with an EC_50_ of 40 nmol L^−1^ compared with 265 nmol L^−1^ for UTP.

## Discussion

Global Ca^2+^ signals in a cell can be classified into brief [Ca^2+^]_i_ transients and sustained [Ca^2+^]_i_ elevation driven by Ca^2+^entry. The findings presented in this study indicate for the first time that iPLA_2_ activity is required for ACE in human keratinocytes. Evidence from the Gross laboratory indicates that iPLA_2_ exists as a ternary complex with Ca^2+^/CaM.^29^ Displacement of CaM from this complex leads to activation of iPLA_2_ which in turn cleaves phospholipids to generate fatty acids such as arachidonic acid and lysoPLs.^5^ Bolotina and colleagues recently demonstrated a role for lysoPLs iPLA_2_ in TG-induced SOCE in rodent cells.^4^ Our results extend their observations by showing that ACE in human keratinocytes also appears to be mediated at least in part by iPLA_2_.

How does iPLA_2_ mediate ACE? In the CIF-iPLA_2_ model of SOCE, the lysoPLs generated by iPLA_2_ are postulated to activate store-operated channels directly.^5^ However, a ternary complex composed of PLCγ, TRPC1 and IP_3_R has been detected in NHEKs.^16^ Although the formation of the complex did not appear to be dependent on store depletion, knockdown of PLCγ or TRPC1 (and TRPC4) suggested a role in Ca^2+^ entry. Could iPLA_2_ participate in the formation or localization of this complex? Recent evidence indicates that PLCγ interacts with the TRPC3 to form a functional intermolecular pleckstrin homology (PH) domain that binds lipids^30^ and that this enhances surface expression of TRPC3 in HEK293 cells. One possibility then is that lysoPLs generated by iPLA_2_ enhance the localization of TRP proteins to the cell surface of keratinocytes, and that inhibition of iPLA_2_ impairs this process. However, we cannot exclude a role for arachidonic acid generated by iPLA_2_ activity, given that it has also been implicated in ACE^31^ although this seems to occur only at submaximal agonist concentrations. Further investigations will be required to delineate the respective functions of lysoPLs and arachidonic acid in Ca^2+^ entry in keratinocytes.

Although none of the agonists used in our experiments induced a sustained [Ca^2+^]_i_ plateau in primary keratinocytes under low (70 μmol L^−1^) [Ca^2+^]_o_ conditions, the [Ca^2+^]_i_ transient induced by LPA under these conditions was significantly longer than that induced by UTP. The duration of a [Ca^2+^]_i_ transient depends on the balance of [Ca^2+^]_i_ extrusion from the cytosol by [Ca^2+^]_i_ pumps (such as the plasma membrane Ca^2+^ ATPase, PMCA) and [Ca^2+^]_i_ re-entry into the ER through SERCA pumps. Both PMCA and SERCA activity may be impaired in NHEK stimulated with LPA, given that LPA has been shown to induce H_2_O_2_ production in HaCaT keratinocytes^32^ and H_2_O_2_ can inhibit PMCA^33^ and SERCA^34^ activity.

When extracellular Ca^2+^ was raised to 1·2 mmol L^−1^, the [Ca^2+^]_i_ peak generated by the application of exogenous nucleotides was followed by an elevated plateau. Importantly, addition of Mn^2+^ to the medium after UTP stimulation led to quenching of the Fluo-4 signal (data not shown), indicating that under these conditions UTP activated Ca^2+^ entry. In contrast to extracellular nucleotides, LPA stimulation did not produce an elevated Ca^2+^ plateau in keratinocytes even in the presence of millimolar levels of [Ca^2+^]_o_ (Fig. 4b). The reason LPA fails to activate ACE in these cells is unclear but studies on Jurkat T cells and a lung fibroblast cell line also found that LPA did not induce Ca^2+^ entry in these cells.^27^,^28^ Thus it appears that despite its ability to mobilize Ca^2+^ from internal stores, LPA does not promote robust Ca^2+^ entry in several distinct cell types. The contrast between UTP and LPA induced [Ca^2+^]_i_ mobilization may be due to differential coupling of LPA receptor activation to STIM1 or Orai1/CRACM1, two newly discovered mediators of SOCE.^35^–^38^ Indeed we have observed that although both UTP and LPA evoke translocation of STIM1 to the PM, the duration of STIM1 localization to the PM is significantly shorter in LPA treated cells.^39^

In conclusion, the work presented here demonstrates that extracellular nucleotides trigger Ca^2+^ release in cultured human keratinocytes, and when external Ca^2+^ is in the millimolar range, Ca^2+^ release follows sustained ACE. Stimulation with LPA also evoked Ca^2+^ release, but without inducing robust Ca^2+^ influx. Pharmacological inhibition of iPLA_2_ impaired ACE, highlighting the importance of iPLA_2_ in ACE in keratinocytes.
